# A Thesis on the “Vis Medicatrix Naturœ,”

**Published:** 1859-01

**Authors:** S. Watkins Vaughan

**Affiliations:** Summerfield, Alabama, for the degree of M. D.


A T L A N T A

### Medical and Surgical Journal.

VOL. IV.] JANUARY, 1859. [No. V.

### ORIGINAL COMMUNICATIONS.

### ARTICLE I.

*A Thesis on the "Vis Medicatrix Naturæ,"* presented to the Faculty of the Atlanta Medical College by S. WATKINS VAUGHAN, Jr., of Summerfield, Alabama, for the degree of M. D. Session of 1858. Published by the Faculty.

As the painter from his picture gallery looks out upon the landscape beneath, he finds much to arrest his gaze and challenge his admiration, and yet each beauty in the lovely picture is to him but the index to brighter views beyond—the beginning of a new out-going to his ever reaching fancy, an endeavor after the end. So with our subject and the votary of medicine; rich fields of thought are here presented, not less inviting to the young physician than the ever varying lines of an Italian sunset to the eye of nature's connoisseur. What tho' its gateway be closed and he shut out for a time from its pleasure grounds of thought, may he not, from a stand point without, glance at its outlined beauties and while he sighs for the luxuries that are beyond, lift up his head and learn that in the *labor vincit omnia* he has a key to its richest delights—the mystic stone with which to pass this golden gate, and enter upon a heritage whose occupants are the sons of science.

But to our subject, the "Vis Medicatrix Naturæ," that strange endowment of the physical man, ever present in sickness, never absent in health, given us as a provision against the encroachment of disease, yet abounding most in ourgreat progenitor Adam, at a time when disease was known not to exist; (albeit with us there is time enough for the shield, buckler and helmet, with the trench dug about our tents, when the enemy has encamped against us); bequeathed by him to us as a sort of privileged body guard or sentry to stand watch on the out works of the system and cry the enemy are upon us, the castle has been taken.

What is it—this proteiform something, which adapts itself so readily to all conditions of the body, (like the nostrum venders of the day) treating all diseases by the same remedy and with surpassing success, until surfeited by the renown thus acquired, becomes ambitious of new achievements and brighter laurels, resolves on a metamorphosis to accomplish these results, assumes the form and garb of disease itself, finds out its secret tenting place, enters the camp in disguise, and after conciliating the friends of the afflicted—especially the man with the saddle-bags—beats out the enemy with his own weapons, carries off the patient, and stands forth to the Medical world the embodied representative of the healing art, the king cure-all of remedies, the "vis medicatrix naturæ." Is it then a specific agent? What is its peculiar office?

We must be brief, as the press of other duties is upon us. If we should venture to differ in our views with those high in authority, we at least shall have been honest in proclaiming it; if charged with irreverence to the great promulgator of the doctrine—the father of medicine himself—we place ourselves behind the opinions of a Dickson and a Rush, and cry they too were heretics.

Firstly then, "it is the power nature possesses in common with medicine of healing" the sick. In medicine, this power is clearly exhibited in the prompt and perfect cure of disease, in the arrest of its fell influence and its removal from the system, thus restoring to the body its needful quantum of health. This may be effected by a direct or an indirect impression, as when opium is applied to an aching tooth and its tortures made to cease; as when quinine is given as an interruptive, and the ague cake is dissolved—its leaven thrown out of the system—or as when a revulsive action is wanted, by the blister on the surface, the indication is fill-ed and the danger to the internal organs averted.

Does nature give us any such demonstrations. Surely not. Ask the poor wretch already disheartened by disease and racked with pain until his sinking energies cry out for help to wait on this vis medicatrix naturæ and he will drive you from his presence with disdain—you would not mock him thus. The glory of our profession has not thus far depart-ed; but do we not, at times witness, the revulsive efforts of this secret agent in the trickish pranks of rheumatism, the capriciousness of gout, as when by metastasis in the latter, from the heels to the heart, the patient goes off in a trice.

Have we not further testimony in the state which some-times obtains through the sympathetic system, whereby the violence of an affection is transferred from the organ pri-marily invaded, to another equally important, and the pa-tient dies from meningitis, that had withstood an attack up-on the womb or other organ primarily involved. Sure-ly, if these are its evidences, the "Medicatrix" at least, is a misnomer, there is nothing here analogous to results which follow the proper administration of medicine. But again, it is ever on the alert as a prophylactic, a preventive of disease.

A grain of sand or other substance falls upon the conjunc-tiva and its sympathies are at once manifested in a copious flow of tears, intended, we are told, to wash out the offend-ing substance and preserve the sight, albeit, the tears flow just as freely, as if the cause had been sufficient, (as from a pointed instrument) at once to obliterate all vision—here, perchance, the grief for a friend departed, is thrown off in the secretion, or rather is not all this the result simply of the mechanical arrangement of the parts, the effect of an in-jury, which if elsewhere applied, had produced very different manifestations. Direct the instrument differently, and let it impinge the walls of the temporal artery and instead of the tears we have an out-gushing of the life blood, driven on by the muscular mechanism of the heart itself, and all the

Vis Medicatrix Naturæ in the world will not arrest the hemorrhage. In the words of one whom we delight to quote, "here comes in the Vis Medicatrix Materia Medicæ, and without it the man will die." The manifestations in either case were but those of life itself, (that great, all pervading, principle, electricity if you like,) the existence of which no one pretends to question. No one, we say, will deny that he lives—that he possesses a principle which presides over his physical organism, imposing upon it laws for the discharge of its multiplied functions, as of use, waste and supply, any one, of which process, is as essential to its well being as the other.

Varnish over the surface of the body for a length of time, lock up its excretory passages, and death will as certainly ensue, as if you should deprive the individual of wholesome food, or cut off his supply of fresh air. The fiat went forth from Jehovah "Dust thou art and unto dust thou shalt return," and never until now, has the moment elapsed that did not furnish the fulfilment and the evidence in each atom turned out from the system, when its office of nutrition had been subserved.

"The condition of life is death." We begin to die the moment we begin to live, and the appearance of corporeal identity presented in us year after year, is only an illusion—man is the action then of the law, governing those processes—use, waste and supply. A slight morbid impression made upon the system—if not renewed—may undergo a gradual effacement, *causa sublata tollitur effectus*, that is the immediate effects, and the system may return to its former condition, though this is not by any means certain, and for the most part is not entirely restored; in proof of this we often witness the sad sequelæ of Typhoid Fever, in lameness, blindness, or deafness, and even imbecility itself. *Asphyxia* continuing for even a brief period, is fatal, no matter how produced, the heart refusing to assume its wonted diastole and systole; how often thus does death result from even a transient interruption of the functions, where the lesion is slight, if any actually exists. The slight concussion from a fall in apoplexy, may produce death, and so instances might be multiplied, instances in which it would seem that any special principle in the organism, whose special function it was to come to the rescue in cases of emergency, would find fit occasion for the display of its power in the preservation of life itself. But no such principle exists. When a cause of disease is introduced into the system, if it be not sufficient to arrest the ordinary processes of elimination, it may be thrown off just as other effete matter, which is constantly making its escape, and which, if unremoved, would itself become an obstruction and a poison—removed, just as an extra blank thrown upon the lottery wheel, will find an outlet with the balance under the revolving action ot the machinery—not that by its presence any new impulse is acquired ; indeed, if there be a difference in its action, the machinery is retarded, and to increase the number of blanks, would be to increase the obstruction until it finally ceased to act; just so with the body, where its natural channels of outlet are occluded, and it is forced to succumb to disease. There is no new agency here at work. Did the advocates of a vis medicatrix naturæ, when thcy would impress us with the extent and efficacy of its virtues, intend simply to enforce the above facts, a single chapter in our Physiology had taught us well the lesson.

There is no rallying here of forces; no onslaught of recruits; no rush of a reserved corps with some Blucher at its head to beat off this common enemy. We cannot even accredit the system with a resistance to disease, certainly with not so much as we find in the stream, to the sapwood on its surface, which will soon sink to form a dam, a permanent barrier to the water whose supply is diminished from above.

In the words of the great Dickson, "The animal body, like an ingenious piece of mechanism, has a tendency to continue the movement, or mode of action, for which it was made, and which, when it began to live it began to carry on." Then, as we would say of the old chronometer, it is an eight day clock—we would say of man, he has his three score and ten years—as of the clock, we would say, it is well made, it is substantial, it runs well—of a man, whose functions are unimpaired, we would say he is strong and healthful. As we would say of the clock that has suffered from a fall, it has lost its balance, and runs badly—we say of man, his system is disturbed by disease, his equilibrium is destroyed. The spring is wound up in the clock, and some sort of irregular action goes on, but it is no longer a time piece. So life is not extinct, while the low muttering of the invalid tells of cerebral congestion, but its objects are entirely forgotten. Show us the "vis medicatrix naturæ" in the one piece of machinery, and we are prepared to receive it in the other. The tinker is applied to with his instruments in the one case, the physician with his revulsives and active antiphlogistics in the other, and the derangements are adjusted in both. Any slight obstruction in either instance might have yielded to the ordinary action of the machinery. As we could not safely pronounce upon the merits of a clock or other machine by the appearance of strength it presented, neither can we infer that the individual who exhibits most marks of health, whose functions are unimpaired, and who therefore would seem to possess this "vis medicatrix naturæ" in its fullest development, is, by any means, the least liable to disease. On the contrary, do we not find the man of full habit, good complexion and free spirits, who of all others enjoys most the good things of life, constantly on the verge of an attack.

The system is here maintained at its ultimatum of force, so that the least drop of excitement is sufficient to turn the scale and make the action run over into disease, and that too, disease of a much more dangerous form, tending much more directly to the destruction of life, than in the case with far less, if you please, of this resistant force—the vis medicatrix naturæ indeed—but for the formulas of our glorious Materia Medica, furnished in its cathartics and other antiphlogistics, death would give us the finale in most cases, and as the brave but deluded victim, who in fancied security had trusted all to a false friend, feels the first thrust of ingratitude aimed at his breast—the *et tu brute* escapes his lips, and his last agony is told. But again, our efforts in the treatment of disease, we are told, must be "imitative," for in its multiplied forms, it is urged, we have but the arrangement of the forces of this Captain-General, with a view to the expulsion of some morbific agent, or renovation of some injured portion of structure. Of course then the nearer we approach in our therapeutical appliances, the operations of this our ally, the more triumphant will be our success, and yet *mirabile dictu*, such is our perverseness, that where the vis medicatrix concentrates his heat, we often resort to cold applications and vice versa, ice to the throbbing temple, bricks or blisters to the extremities when the temperature is lowered and our efforts—stranger still—save the patient. Where by the action of this agent, the system is impoverished, the blood defibrinated or otherwise robbed of its nutritive properties, we, with our iron, generous diet and cod-liver oil, would seek to build it up, and here, if time permitted, we might enlarge on this very interesting part of the subject.

How opposite the conditions which obtain in a part when it suffers from the effects of a recent injury, or becomes the seat of chronic ulceration. Our efforts to overcome these conditions are not less diligent in the one case than the other, for either if unarrested, might prove fatal, and yet in both, we witness *but* the operation of the "vis medicatrix naturæ."

With us, at least, there is nothing harder than to reconcile that doctrine with itself, which, while it regards inflammation as a healthy process; fights it from the outset with all the antiphlogistics available, the position, as the enigma has it, is not unlike that of the human family in regard to a certain familiar article of house furniture, the bed, "which no one desires to be without, yet no one wishes to keep."

Some Pathologists, says Dickson, teach that disease consists merely in an increase or enhancement of the natural actions of the body, or the reverse; in other words, that morbid differs from natural action in degree only, or intensity. This is an important and mischievous error. There is *perversion*, not simply difference in force—essential difference in nature, or kind of action, as well as in its degree. This is proved by the changes which take place in the fluids. The blood, the secretions, the effused lymph and pus becoming specially contagious, and also by the peculiarities of disease, solids built up, as in fungus hæmatodes, tubercle osteo sarcoma and cancer.

